# Multistage Porous Carbon Derived from Enzyme-Treated Waste Walnut Green Husk and Polyethylene Glycol for Phase Change Energy Storage

**DOI:** 10.3390/ma17061379

**Published:** 2024-03-18

**Authors:** Ziming Wang, Luo Liu, Hui Cao

**Affiliations:** College of Life Science and Technology, Beijing University of Chemical Technology, Beijing 100029, China; 2022201317@buct.edu.cn

**Keywords:** phase-change materials, thermal energy storage, walnut green husk, enzyme treatment

## Abstract

The thermal storage performance, cost, and stability of phase-change materials (PCMs) are critical factors influencing their application in the field of thermal energy storage. Porous carbon, with its excellent support, thermal conductivity, and energy storage properties, is considered one of the most promising support matrix materials. However, the simple and efficient synthesis of high-performance and highly active bio-based materials under mild conditions still faces challenges. In our work, a novel method for preparing new functional composite phase-change materials based on enzyme treatment technology and using waste walnut green husk biomass and polyethylene glycol as raw materials was developed. The enzymatic treatment method exposes the internal structure of the walnut green husk, followed by the adjustment of the calcination temperature to increase the adsorption sites of the biochar, thereby stabilizing polyethylene glycol (PEG). The porous properties of walnut green husk biochar effectively regulate the phase-change behavior of polyethylene glycol. In the biochar carbonized at 600 °C, the PEG loading reached 72.09%, and the absorption heat of the solid–solid phase-change material (SSPCM) reached 194.76 J g^−1^. This work not only enriches the application of biomass in heat storage but also demonstrates the broad prospects of SSPCMs in solar thermal utilization.

## 1. Introduction

With the development of the social economy, the demand for energy by humanity is increasing. In response, efforts are underway to explore green and renewable energy sources to replace Earth’s depleting resources, such as solar energy, wind energy, geothermal energy, and more. However, the intermittent nature of these energy sources poses significant challenges to their efficient utilization, making effective energy storage crucial [1,2,3]. Currently, in the field of thermal energy, despite the widespread use of sensible heat for storage, its limitations, such as low heat storage capacity, non-constant release of heat, and bulky storage devices, have hindered further applications [4]. Therefore, the exploration of an economically viable energy storage method has garnered global attention, and the discovery of latent heat storage methods has the potential to address the shortcomings of sensible heat storage.

Phase-change materials (PCMs) [5,6,7] that store and utilize the heat released or absorbed during phase transitions can achieve efficient energy conversion, addressing issues such as energy supply–demand imbalances and insufficient energy reserves, thereby improving energy utilization efficiency. PCMs are considered an ideal solution for the future development of new energy sources. However, the tendency of single-phase-change materials to leak during phase transitions leads to low energy storage efficiency. The use of porous carriers with capillary forces and surface tension for strong adsorption on phase-change cores forms shape-stable phase-change materials (SSPCMs), fundamentally addressing this issue. In recent years, a variety of porous carriers have been applied, including carbon nanotubes (CNTs) [8], graphene oxide (GO) [9,10], Mxene [11], and foam metals [12,13], but difficulties in the preparation processes and cost issues have limited their widespread application.

Recently, biomass, such as sugar cane [14], mushroom [15], coffee [16], hazelnut wood [17], grounds [18], and walnut [19], as a bountiful renewable resource in nature, has become more attractive in the preparation of useful carbonaceous materials, which are a type of carbon material prepared from biomass and have the advantages of being renewable, environmentally friendly, and low-cost. These materials are typically derived from plants, microorganisms, or other organisms, using a series of processing steps to convert biomass into carbon materials. They are widely used in environmental governance, energy, material science, environmental protection, building insulation, and other sustainable development fields. In contrast to other porous carriers such as CNTs and GO, utilizing waste biomass to prepare porous carriers effectively addresses the challenge of high costs. Moreover, these carriers are renewable and possess tunable porosity, high biocompatibility, and structural stability, making them highly anticipated for applications in the field of phase-change energy storage. Wan et al. [20] prepared a thermally stable SSPCM by impregnating pinecone biochar with palm stearin, achieving a phase-change latent heat of 84.74 J g^−1^ and a high thermal conductivity of 0.3926 W (m^−1^·K^−1^). Liu et al. [21] demonstrated that the PEG/CSBC composite material obtained from corn stover biochar (CSBC) and polyethylene glycol (PEG) exhibited a high latent heat of 100.2 J·g^−1^, emphasizing the critical role of the biochar’s porous structure and surface activity in influencing the biochar’s energy storage performance. Recent research by Kim et al. [22] indicated that biochar prepared from different biomass sources has distinct structural properties, leading to significant differences in the loading–storage capacity of organic phase-change materials. They highlighted the crucial role played by surface functionality, structural characteristics, bio-material type, molecular interactions between PCMs and biochar, and pyrolysis temperature in determining the thermal performance of the fabricated FSPCC. Based on this analysis, biomass porous carriers have extensive potential applications in heat storage, and factors such as temperature, modifying agents, biomass type, and surface activity can affect structural properties and loading performance [23].

Porous carbon, with excellent support, thermal conductivity, and energy storage properties, is considered one of the most promising support matrix materials, surpassing other materials and porous polymers [24,25,26,27,28]. Sun et al. [29] synthesized a novel paraffin-separated porous carbon (PW/HPC) SSPCM. The large surface area of HPC allows for a substantial PW load. The introduction of synthesized carbon materials increased thermal stability and improved heat absorption capacity, resulting in a thermal conductivity of PW/HPC 1.8 times higher than that of pure PW. Wang et al. [30] discovered that the capillary force and hydrogen bonding between polyethylene glycol (PEG) and porous carbon carriers in PEG/CSBC composite materials lead to a higher thermal conductivity of SSPCM while preventing leakage during the solid-to-liquid phase transition. Therefore, the excellent properties of carbon materials make them an attractive candidate for SSPCM synthesis, and the application of porous carbon-based SSPCMs in buildings will reduce indoor temperature fluctuations, thus achieving energy savings [31]. This is especially due to the composition of the walnut green husk (high lignin–fiber content), which is a good precursor for the formation of micropores and mesopores during carbonization and activation to obtain a large pore volume and a high specific surface area [32,33,34]. However, bio-based carbon materials also face many problems, such as complex synthesis routes, the preparation process requiring a certain temperature and pressure, and the performance still needing to be improved [35,36,37]. The simple and efficient synthesis of high-performance and highly active bio-based materials under mild conditions still faces challenges.

This study proposes, for the first time, the co-pyrolysis of waste walnut green husk biomass at 600 °C to obtain porous carbon as a matrix, with ligninase as a modifier and polyethylene glycol as a composite material, to prepare a multifunctional composite phase-change material with heat storage and adsorption properties. This biomass source is clean and readily available, and the resulting porous carrier (PC) exhibits good support and loading effects on PEG, thereby enhancing heat storage and thermal conductivity. The application of this composite phase-change material in building materials will significantly increase the efficiency of clean energy utilization and promote economic development.

## 2. Experimental Procedure

### 2.1. Materials

The shape-stable phase-change material was prepared using waste walnut green husk (WGH) as the biomass source. The walnut green husk was then washed with deionized water and dried to a completely dry state in a 60 °C oven to avoid uneven heating during co-pyrolysis. PEG2000 was considered the latent heat storage material. All other chemical reagents used were of analytical grade.

### 2.2. Preparation of PC (Porous Carbon)

**Figure 1:**
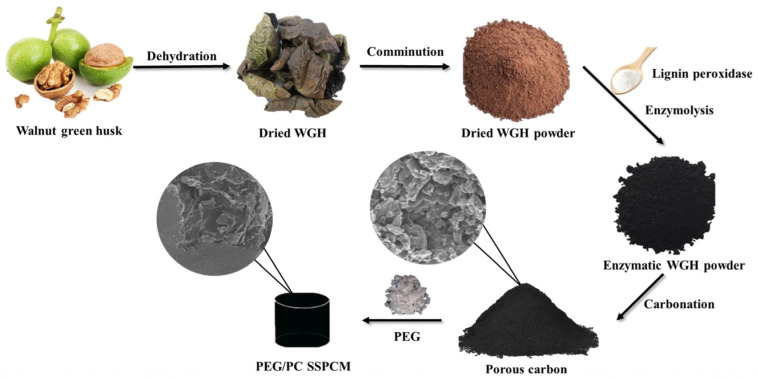
Schematic diagram of the preparation process for PC and PEG/PC SSPCM.

The process for preparing porous carbon is illustrated in Figure 1. Firstly, the dried walnut green husk was ground into a powder. Subsequently, it was mixed with ligninase at a mass ratio of 10:1 and allowed to stand at 50–60 °C for 24 h to obtain modified walnut green husk. The modified walnut green husk was then placed in a tubular furnace and heated to 600 °C at a rate of 5 °C/min under a nitrogen atmosphere. After maintaining this temperature for 2 h, porous carbon (PC) was obtained.

### 2.3. Preparation of PEG/PC SSPCM

The preparation process of PEG/PC SSPCM is illustrated in Figure 1. With reference to previous works in related fields and in order to facilitate single-variable analysis, we fixed the mass ratio of porous carbon (PC) and polyethylene glycol (PEG) at 7% [38]. In detail, 0.7 g of PC prepared from walnut green husk is combined with 10 g of PEG in a beaker and heated in a 60 °C water bath until complete melting. Subsequently, PEG/PC SSPCM is prepared using a vacuum impregnation method, placing it in a 60 °C constant-temperature vacuum drying oven for 2 h of vacuum impregnation. This process is repeated three times until the sample reaches a stable mass and the pore structure is saturated. After completion, the sample is taken out, resulting in the preparation of PEG/PC SSPCM composite phase-change energy storage material.

### 2.4. Characterization Methods

Scanning electron microscopy (SEM): SEM was employed to observe the microstructure of the porous carbon (PC) obtained after modification with ligninase and the structure and morphology of PEG/PC SSPCM prepared by vacuum impregnation. A scanning electron microscope (JEOL, Tokyo, Japan, JSM-7800F) was utilized, and a thin layer of gold film was coated on the surfaces of PC and PEG/PC SSPCM before observation.

Brunauer–Emmett–Teller (BET) Surface Area Analysis: The specific surface area and pore size distribution of the biomass porous carbon (PC) material were characterized using the Brunauer–Emmett–Teller method.

X-ray Diffraction (XRD): XRD was employed to study the crystal structure and chemical compatibility of PEG, PC, and PEG/PC SSPCM. Scans were performed in the 2θ range of 5° to 70°, with a scan rate of 2°/min, using Cu Kα radiation.

Fourier-Transform Infrared Spectroscopy (FTIR): FTIR (SHIMADZU, Japan, IRTACER-100) was utilized to evaluate the chemical compatibility of PC and PEG/PC SSPCM.

Differential Scanning Calorimetry (DSC): DSC (TA, USA, DSC25) was used to investigate the phase-change behavior of PEG and PEG/PC SSPCM under a nitrogen atmosphere, with a heating rate of 5 °C/min from 40 °C to 75 °C.

Thermogravimetric Analysis (TGA): TGA (NETZSCH, Selb, Germany, TG 209 F3) was employed to study the thermal stability and decomposition performance of PEG and PEG/PC SSPCM. Samples were scanned in the temperature range of 50 to 600 °C, with a heating rate of 10 °C/min, under a nitrogen atmosphere at 20 mL/min.

Leakage Assessment: The thermal stability of PEG/PC SSPCM and PEG was determined by assessing the leakage of the materials at different recorded points from 25 °C to 80 °C.

Thermal Infrared Imaging: Compare the color brightness of PEG and PEG/PC SSPCM with changing temperatures to determine the heat storage characteristics of the materials.

## 3. Results and Discussion

### 3.1. Structure of PEG/PC SSPCM

**Figure 2:**
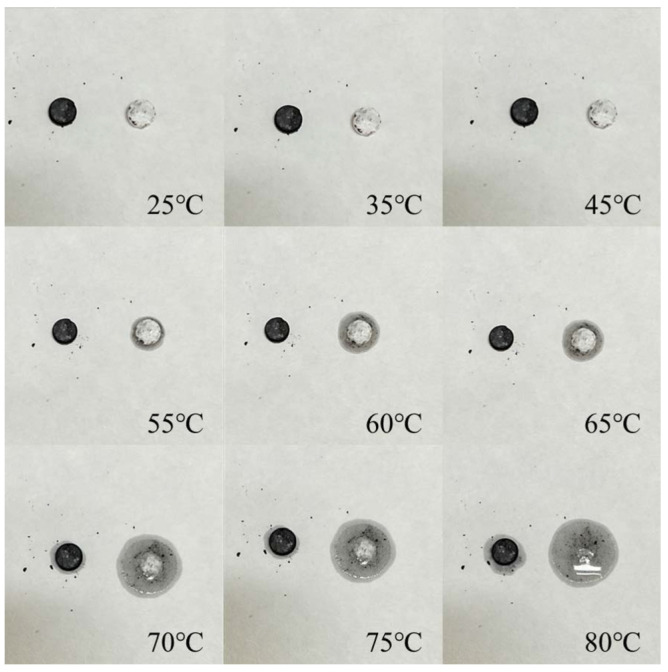
Shape stability experiment of PEG and PEG–porous carbon SSPCM.

To evaluate the shape stability of the composite materials, leakage tests were conducted. The leakage conditions of PEG and PEG–porous carbon SSPCM from 25 °C to 80 °C are shown in Figure 2. Taking 0.05 g of PEG and PEG–porous carbon SSPCM, the two materials were pressed into small chips, and leakage was observed at temperatures ranging from 25 °C to 80 °C. The black chips represent PEG–porous carbon SSPCM, while the gray–white chips represent PEG.

From Figure 2, it can be observed that before 45 °C, the surface shapes of both PEG and PEG/PC SSPCM did not show significant changes. As the temperature increased to 55 °C, slight leakage occurred with PEG, while the morphology of PEG/PC SSPCM remained unchanged. At 70 °C, PEG melted more, but incomplete melting was observed, resulting in minimal leakage for PEG/PC SSPCM. When the temperature reached 75 °C, complete melting of PEG was observed, with PEG/PC SSPCM showing a small amount of leakage. Even at 80 °C, the morphology of PEG/PC SSPCM remained relatively intact. We proposed that PEG fills the micropores and pore structure of porous carbon to form a uniform composite structure, which was confirmed by SEM images. This filling effect can reduce the penetration of liquid or gas through the composite material, thereby reducing the amount of leakage [39,40].

**Figure 3:**
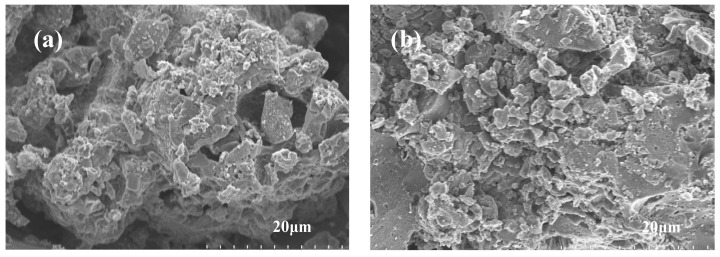
SEM images of PC prepared with (**a**) and without (**b**) ligninase hydrolysis modification.

The SEM image of PC is presented in Figure 3. It can be observed that there are some carbon frameworks within PC. Furthermore, comparing Figure 3b after ligninase hydrolysis modification with Figure 3a (unmodified PC), it is evident that the unmodified PC has fewer micro-mesoporous structures in its pore network, whereas the modified PC exhibits a significant increase in micro-mesoporous structures. Combined with BET data, we quantitatively characterized the pore volume sizes of the two materials at different scales (mesopore volume), as follows: PC < modified PC. This is consistent with the conclusion of the SEM patterns. A similar conclusion was also reached by Wang et al. [30]. The presence of more micro-mesoporous structures in porous carbon can significantly increase the surface area available for interactions with PEG, which offers more sites for adhesion or absorption of PEG molecules. Specifically, micro-mesoporous structures, due to capillary phenomena, enable the loading of more PEG and secure adsorption, resulting in increased energy storage capacity.

**Figure 4:**
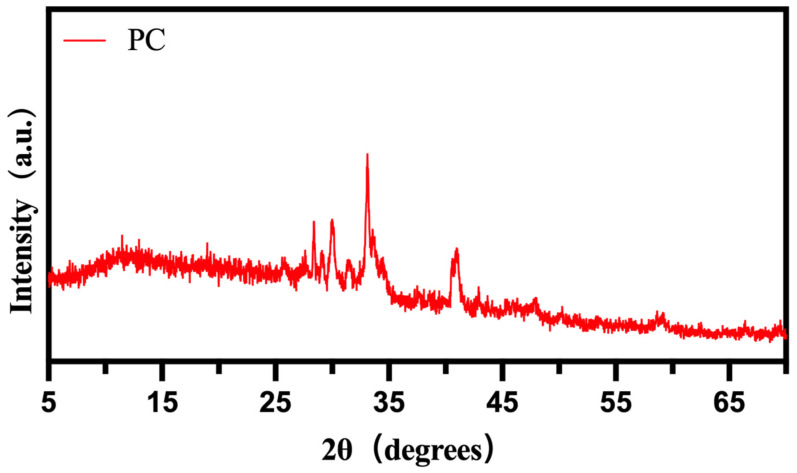
XRD spectrum of PC.

The XRD spectrum of PC is presented in Figure 4. The PC sample exhibits a diffraction peak at 13.62° (2θ), corresponding to the (001) crystal plane, indicating the presence of an amorphous phase in the bio-carbon carrier. Additionally, prominent diffraction peaks are observed at 41.3° and 43.9° (2θ), corresponding to the (100) and (002) crystal planes, respectively. Furthermore, there are strong diffraction peaks at 34.72° and 40.51° (2θ), corresponding to the (020) and (021) crystal planes, respectively. These findings suggest the coexistence of amorphous and crystalline phases in the prepared PC carrier [41,42].

**Figure 5:**
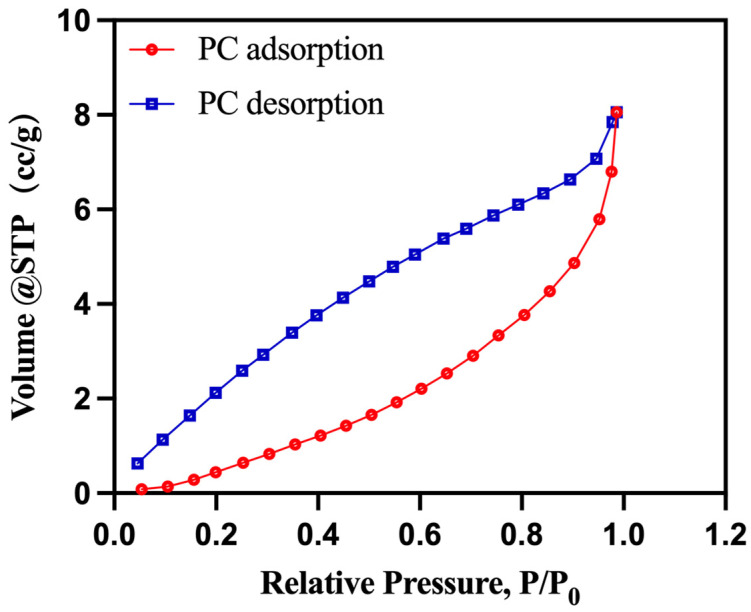
Nitrogen adsorption–desorption isotherm of PC.

The specific surface area and pore size distribution of PC were determined using the Brunauer–Emmett–Teller (BET) method. The nitrogen adsorption–desorption isotherm is shown in Figure 5. The quantity of the adsorbed gas increased, exhibiting a hysteresis curve in a wide range of relative pressure. When P/P0 is less than 0.4, the adsorption curve increases with the nitrogen adsorption, indicating the dominance of micropores at this stage. When P/P0 is in the range of 0.4–0.9, there is a significant increase in nitrogen adsorption, and the adsorption curve rises rapidly, indicating the prevalence of mesopores. When P/P0 is in the range of 0.9–1.0, nitrogen adsorption continues to increase rapidly, suggesting the dominance of macropores.

**Figure 6:**
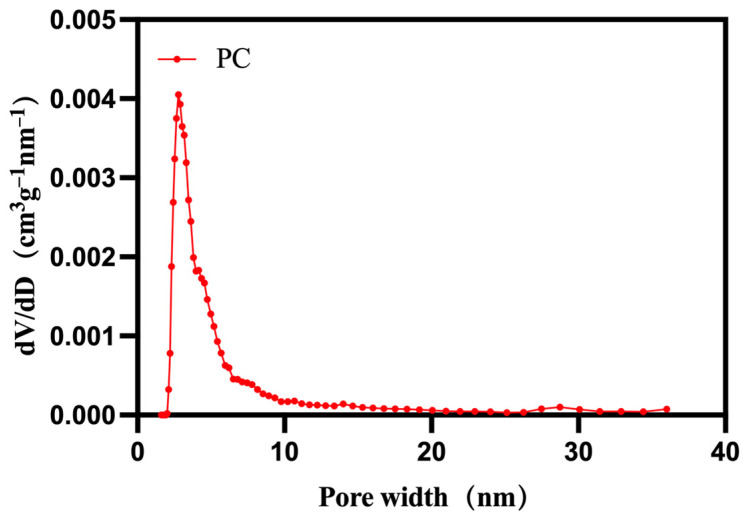
Pore size distribution of PC.

Figure 6 illustrates the pore size distribution of PC. It can be observed that the bio-carbon prepared by this method has a rich micro-mesoporous structure with almost no microporous structure. The pore size distribution is mainly concentrated around 0.0041 cm^3^·g^−1^. In conclusion, this bio-carbon exhibits diverse micro- and mesoporous structures, facilitating better material loading. Additionally, it is evident that the modifying agent has a significant impact on the pore structure of the prepared bio-carbon [40].

**Figure 7:**
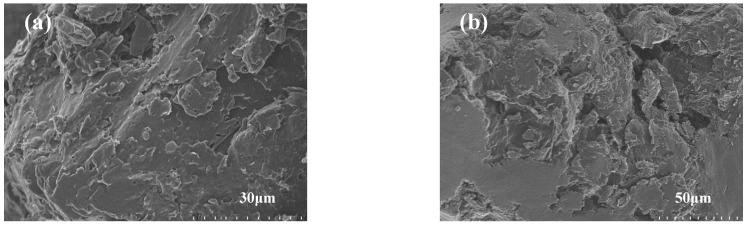
Electron microscopy images of PEG/PC SSPCM magnified to 30 µm (**a**) and 50 µm (**b**).

The electron microscopy results indicate that PEG/PC SSPCM has a compact morphology. At different magnifications (Figure 7), PEG almost occupies all the seamless pores, indicating the relatively sufficient load-bearing capacity of PEG. Additionally, the stable carbon framework of PC can inhibit the leakage of PEG, contributing to better shape stability. This proves that vacuum impregnation is an effective loading method. This experimental conclusion is consistent with the experimental results of the leakage test mentioned in Figure 2. It also demonstrates the comprehensive combination of PC prepared from green husk as the raw material with ligninase as a modifying agent in the phase-change medium. 

**Figure 8:**
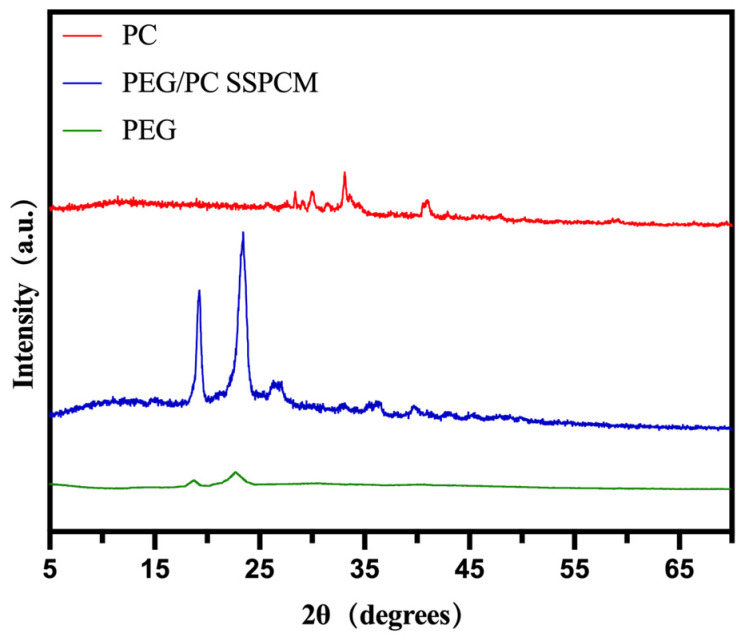
XRD spectra of PEG, PC, and PEG/PC SSPCM.

Observing the XRD spectrum of PEG/PC SSPCM (Figure 8), obvious diffraction peaks appeared at 19.06° and 23.20°. By comparing the XRD spectra of pure PEG and PEG/PC SSPCM, it is obvious that both pure PEG and PEG/PC SSPCM exhibit similar peaks, and through reference [43], the crystal plane index at these peaks should correspond to (120) and (112). However, the peak of the sample shows an obvious high-angle shift after compounding. This indicates that compressive stress or a defective structure may have been generated after the introduction of PC. At the same time, some new peaks were generated around 27 degrees, proving that new interactions occurred. In summary, the PC carrier was successfully loaded with PEG using the chemical loading method.

**Figure 9:**
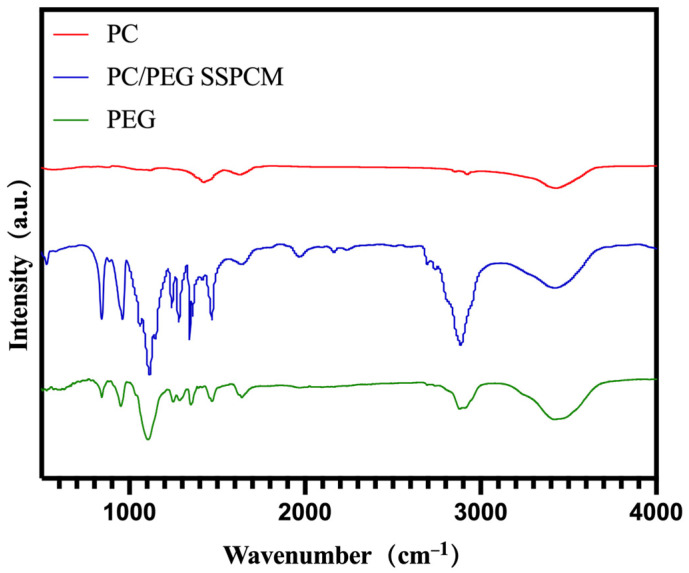
PEG, PC, and PEG/PC SSPCM Fourier-Transform Infrared Spectroscopy (FTIR) spectra.

To further demonstrate the different intermolecular interactions, FTIR tests were performed. In the FTIR spectrum of PC, the peak around 600 cm^−1^ indicates the planar bending vibration of C-C (Figure 9), which is one of the typical functional groups in porous carbon. The peak around 900 cm^−1^ typically represents the planar bending vibration of C-H. The peak around 1100 cm^−1^ indicates the stretching vibration of C-O, possibly originating from functional groups such as cellulose and lignin in biomass carbon. The peak around 1400 cm^−1^ is usually associated with the stretching vibration of C-H, involving functional groups such as branched alkanes and aromatic compounds. The peak around 1600 cm^−1^ is attributed to the asymmetric stretching vibration of C=C bonds in aromatic compounds. The peak around 2900 cm^−1^ reflects the stretching vibration of C-H in aliphatic compounds (e.g., fatty acids, glycerol) in biomass carbon. The peak around 3400 cm^−1^ is often related to the stretching vibration of O-H or N-H functional groups, indicating the presence of water molecules.

In the FTIR spectrum of PEG/PC SSPCM, the peak around 800 cm^−1^ represents the bending vibration of C-H, likely indicating aliphatic structures in both biomass carbon and polyethylene glycol (PEG). The peak around 900 cm^−1^ represents the bending vibration of C-H and O-H, possibly involving functional groups such as -O-CH_2_- and -O-CH_3_ in PEG, as well as hydroxyl groups (OHs) and C-O-C in biomass carbon. The peak around 1150 cm^−1^ represents the stretching vibration of C-O and C-O-C, involving hydroxyl groups and ketone groups in PEG and biomass carbon. The peak around 1400 cm^−1^ represents the asymmetric stretching vibration of CH_2_, typically originating from aliphatic structures in PEG. The peak around 1500 cm^−1^ represents the stretching vibration of C=C, possibly due to the presence of polycyclic aromatic compounds in biomass carbon. The peak around 1700 cm^−1^ represents the stretching vibration of C=O, likely originating from a large number of ketone groups in PEG and biomass carbon. The peak around 2000 cm^−1^ is generally associated with the stretching vibration of C=C and C≡C bonds, although infrared absorption peaks are rarely observed in this range. The peak around 2250 cm^−1^ is seldom seen and is typically related to the stretching vibration of C≡C bonds. The peak around 3400 cm^−1^ is commonly associated with the stretching vibration of O-H and N-H, often indicating the presence of water molecules in phase-change materials.

It can be observed that, although PEG/PC SSPCM exhibits characteristic absorption peaks similar to those of PEG and PC, some new peaks appeared that were different from those of the individual substances. Through analysis, it can be preliminarily concluded that the loading process of PC and PEG in the impregnation is a chemical method.

### 3.2. Thermal Energy Storage Properties of PEG/PC SSPCM

**Figure 10:**
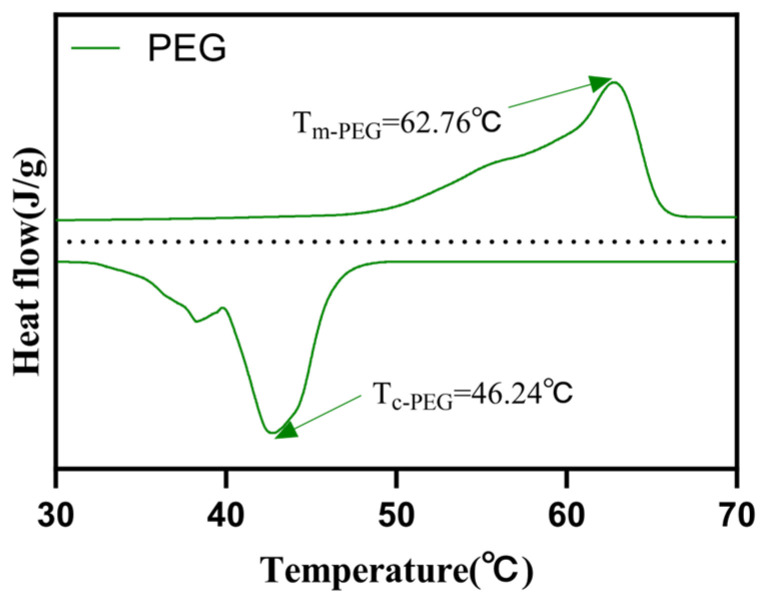
Differential Scanning Calorimetry (DSC) curve of PEG.

Thermal storage performance is a key factor in expanding the application of phase-change materials. From the DSC curve of PEG in Figure 10, it can be observed that the melting point of PEG is at 62.76 °C, with a solidification point of 46.24 °C. By integrating the enthalpy peak, the phase-change enthalpy of PEG is determined to be 270.16 J g^−1^.

**Figure 11:**
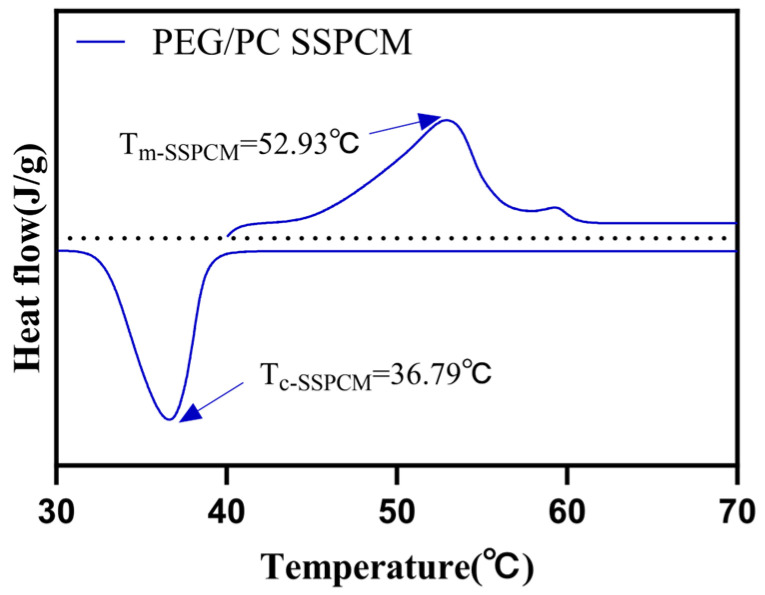
Differential Scanning Calorimetry (DSC) curve of PEG/PC SSPCM.

In Figure 11, the DSC curve of PEG/PC SSPCM shows that the phase-change temperature range of the composite material is approximately between 36 °C and 53 °C. Comparing the temperature ranges of PEG and PEG/PC SSPCM, it is evident that the addition of PC reduces the temperature range of PEG, decreases the degree of undercooling (allowing the phase-change material to store more heat during melting), and releases more heat when solidifying into large crystals [44]. PEG/PC SSPCM shows better performance for the following reasons: First, PC has a large number of micropores and pore structures, which provides a large number of nucleation sites and promotes the formation of crystal nucleation during the phase change of PEG. This helps reduce the occurrence of supercooling and enables phase change even at lower temperatures, thus extending the effective temperature range of phase-change materials [45]. Second, PC has excellent thermal conductivity and can effectively promote heat conduction during phase change. This helps accelerate the phase-change process, allowing the phase-change material to reach an equilibrium state faster and reducing the occurrence of supercooling [43]. Third, the addition of PC can increase the total heat capacity of phase-change materials. PC itself has a high heat capacity. After adding it, it can increase the total mass and heat capacity of the phase-change material, causing it to absorb or release more heat during the phase-change process, thus improving its heat storage and release capabilities. The corresponding phase-change parameters, including melting enthalpy (Δ*H_m_*), crystallization enthalpy (Δ*H_c_*), melting temperature (*T_m_*), and crystallization temperature (*T_c_*), are presented in Table 1.

To evaluate the impact of PC on the latent heat of phase change and investigate the loading situation of PEG/PC SSPCM, the data presented above were utilized to study the loading rate and relative enthalpy efficiency of PEG/PC SSPCM. Conclusions were drawn from the data of Equations (1) and (2) to explore the mechanism between the microstructure and the performance of SSPCM.

(1) $$R=\frac{\Delta H_{m-SSPCM}}{\Delta H_{m-PEG}}\times 100\%$$

(2) $$E=\frac{\Delta H_{m-SSPCM}+\Delta H_{c-SSPCM}}{\Delta H_{m-PEG}+\Delta H_{c-PEG}}\times 100\%$$

In the above Equations (1) and (2), *R* represents the effective loading amount of the material, Δ*H_m-SSPCM_* and Δ*H_c-SSPCM_* represent the melting enthalpy and crystallization enthalpy of the phase-change material, Δ*H_m-PEG_* and Δ*H_c-PEG_* represent the melting enthalpy and crystallization enthalpy of PEG, *R* represents the effective loading amount, and *E* represents the actual percentage of energy storage–release capability in the composite phase-change material, i.e., the effectiveness of latent heat storage. SSPCM represents the composite phase-change material. The data in Table 2 can be obtained through calculations. 

In summary, the loading rate of the PEG/PC SSPCM composite phase-change material reached 72.09%, demonstrating optimal loading effects. The significantly high loading rate ensures the material’s high latent heat performance. The addition of PC enhances the enthalpy value of PEG, as PC’s micro- and macroporous structure allows for more PEG loading, simultaneously increasing thermal conductivity and facilitating rapid enthalpy release.

### 3.3. The Thermal Stability of PEG/PC SSPCM

**Figure 12:**
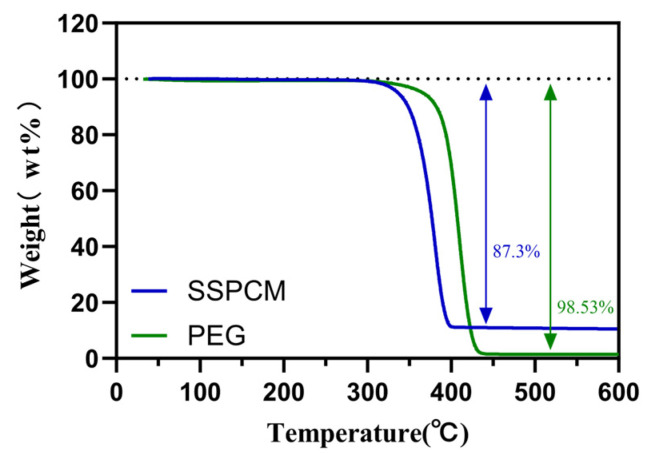
PEG and PEG/PC SSPCM TGA curves.

**Figure 13:**
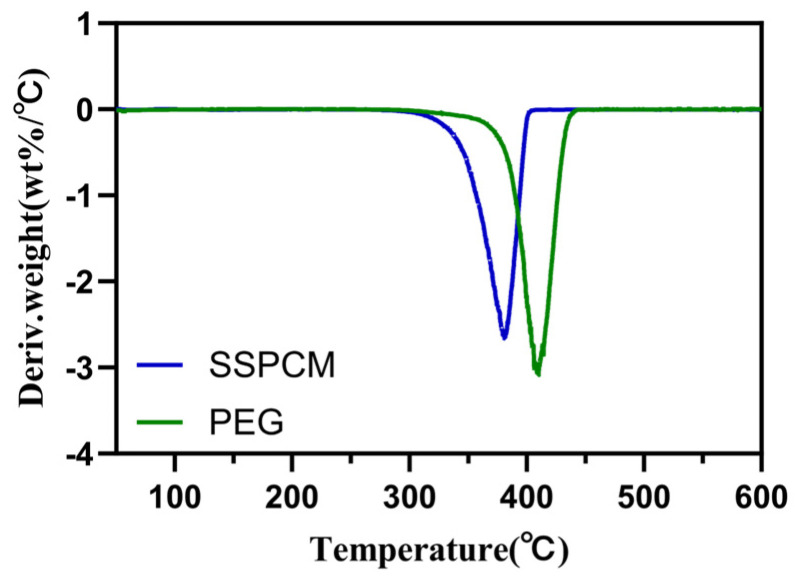
PEG and PEG/PC SSPCM DTG curves.

In Figure 12 and Figure 13, the Thermogravimetric Analysis (TGA) and differential thermogravimetric (DTG, which is obtained by differentiating the TGA curve) curves of PEG and PEG/PC SSPCM are presented. PEG loses 98.53 wt%, leaving 1.47 wt% of residual mass. In comparison, PEG in the PEG/PC SSPCM phase-change material begins to decompose at 306.3 °C. When the temperature reaches 373.1 °C, the decomposition rate is the fastest, and it completely decomposes at 399.4 °C, losing 87.3 wt%, with 13.7 wt% residual mass remaining. Combining this information, it is evident that the decomposition temperature of PEG/PC SSPCM is lower than that of PEG. This is likely due to the increased thermal conductivity provided by the loaded bio-carbon and the good encapsulation of polyethylene glycol [46,47]. This demonstrates the good thermal stability and potential energy storage application prospects of PEG/PC SSPCM.

### 3.4. The Waste Heat Recovery Behavior of PEG/PC SSPCM

Waste heat recovery behavior was investigated by comparing the variations in brightness with respect to temperature of the PEG and PEG/PC SSPCM phase-change materials. Samples of 0.05 g of PEG and PEG/PC SSPCM phase-change materials were compressed into pellets, placed on an electric heating plate, and subjected to a temperature increase from 25 °C to 75 °C in 5 °C increments, with a 5 min hold at each temperature. Thermal infrared images were captured using a handheld thermal infrared imager, imported into a computer, and analyzed to assess the energy storage capacity of the materials.

**Figure 14:**
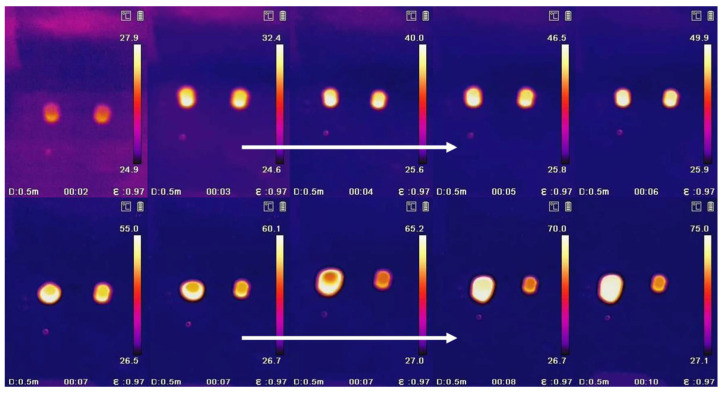
Thermal Infrared Imaging experiment of PEG and PEG/PC SSPCM.

In Figure 14, the left side represents PEG phase-change materials, and the right side represents PEG/PC SSPCM phase-change materials. From left to right, the images illustrate the energy storage behavior of PEG and PEG/PC SSPCM phase-change materials during the heating process.

Ignoring the influence of external environmental temperature, it can be observed that with temperature variations, PEG exhibits a rapid increase in brightness, indicating relatively poor energy storage capacity. In contrast, the PEG/PC SSPCM phase-change material shows a sustained increase in brightness. This suggests that the PEG/PC SSPCM phase-change material, derived from walnut green husk (WGH) modified with ligninase and prepared using porous carbon (PC) as the matrix, has superior energy storage capacity compared to the phase-change medium.

In comparison with other studies, Zhang et al. [48] investigated the thermal performance of LA-SA–carbonized corn core composite PCMs prepared by vacuum impregnation. Gondora et al. [49] utilized a rice husk char emulsion with hexadecane as the core to prepare microencapsulated phase-change materials (MEPCMs). The research article by Hekimoğlu et al. [50] applied a eutectic mixture of lauric acid and capric acid into the framework of activated carbon derived from apricot kernel shells to make PCMs. Kalidasan B et al. [51] synthesized a kind of 3D coconut shell biochar–polyethylene glycol composite as thermal energy storage material. However, few works have used enzymatic treatments in the preparation of phase-change materials. Zhai et al. [52] pointed out that the enzymatic method exhibited excellent performance during the synthesis process, while the chemical method caused more by-products and higher energy consumption.

Inspired by the corresponding result, in our work, the high-efficiency enzymatic treatment process was used for PEG/PC SSPCM synthesis for the reason that the enzymatic treatment of dry biomass, especially waste walnut green peel biomass, can bring some important advantages and effects, such as the degradation of complex biomass structures, the release of valuable compounds, the increase in the yield of enzymatic hydrolysis products, the improvement in the treatability of biomass, and the reduction in environmental impact during biomass treatment. At the same time, the biologically based raw materials—available and clean waste walnut green husk biomass was used as the raw material, and ligninase was employed as a modification method to prepare a low-cost, structurally stable porous carrier—and the mild synthetic process conditions make it a more sustainable option than other types of materials. As a result, the prepared materials have shown good phase-change energy storage properties. The physical loading of PEG onto PC using vacuum impregnation resulted in PEG/PC solid–solid phase-change materials (SSPCMs). The enthalpy of absorption (ΔH) reached 194.76 J g^−1^, with a loading rate of 72.09%, demonstrating excellent structural stability and energy storage capacity.

## 4. Conclusions

In conclusion, for the first time, a novel method for preparing new functional composite phase-change materials with heat storage and adsorption properties via enzyme treatment technology and using waste walnut green peel biomass and polyethylene glycol as raw materials was developed. The analysis of the shape stability, electron microscopy, and BET tests revealed that PC prepared through ligninase enzymatic modification had more micro- and mesoporous structures. It served as an excellent supporting material, effectively loading PEG while maintaining good shape stability and retaining its integrity at 80 °C. The as-prepared PEG/PC SSPCM materials exhibited excellent phase-change heat storage potential, which had an enthalpy of absorption (Δ*H*) reaching 194.76 J g^−1^, with a loading rate of 72.09%, as determined by DSC analysis. Thermal Infrared Imaging shows that the prepared material exhibits good thermal stability and waste heat recovery performance. This new preparation method and phase change bring a new idea, and the corresponding materials are expected to be widely used in the fields of energy storage and conversion.

## Figures and Tables

**Table 1:** DSC data table for composite phase-change material with modified bio-carbon as the matrix.

Samples	*T_m_*/°C	*T_c_*/°C	∆*H_m_*/(J g^−1^)	∆*H_c_*/(J g^−1^)
PEG	62.76	46.24	270.16	213.73
PEG/PC SSPCM	52.93	36.79	194.76	155.77

**Table 2:** DSC data table of composite phase-change material with modified biochar as the matrix.

Samples	*R*/%	*E*%
PEG/PC SSPCM	72.09	72.44

## Data Availability

Data are contained within the article.

## Abbreviations

Abbreviation	Full name
WGH	walnut green husk
PC	porous carbon
PEG	polyethylene glycol
PCM	phase-change material
SSPCM	solid–solid phase-change material
